# 5-Aminolevulinic acid antiviral efficacy against SARS-CoV-2 omicron variant in vitro

**DOI:** 10.1186/s41182-022-00422-7

**Published:** 2022-04-27

**Authors:** Mya Myat Ngwe Tun, Takaya Sakura, Yasuteru Sakurai, Yohei Kurosaki, Daniel Ken Inaoka, Norifumi Shioda, Chris Smith, Jiro Yasuda, Kouichi Morita, Kiyoshi Kita

**Affiliations:** 1 Department of Virology, Institute of Tropical Medicine (NEKKEN), Nagasaki University, 1-12-4 Sakamoto, Nagasaki, 852-8523 Japan; 2 Department of Molecular Infection Dynamics, Shionogi Global Infectious Diseases Division, Institute of Tropical Medicine (NEKKEN), Nagasaki University, 1-12-4 Sakamoto, Nagasaki, 852-8523 Japan; 3 Department of Emerging Infectious Diseases, Institute of Tropical Medicine (NEKKEN), Nagasaki University, 1-12-4 Sakamoto, Nagasaki, 852-8523 Japan; 4 National Research Center for the Control and Prevention of Infectious Diseases, Nagasaki University, 1-12-4 Sakamoto, Nagasaki, 852-8523 Japan; 5 Department of Genomic Neurology, Institute of Molecular Embryology and Genetics (IMEG), Kumamoto University, Kumamoto, Japan; 6 Graduate of School of Pharmaceutical Sciences, Kumamoto University, Kumamoto, Japan; 7 School of Tropical Medicine and Global Health, Nagasaki University, 1-12-4 Sakamoto, Nagasaki, 852-8523 Japan; 8 Department of Host-Defense Biochemistry, Institute of Tropical Medicine (NEKKEN), Nagasaki University, 1-12-4 Sakamoto, Nagasaki, 852-8523 Japan

**Keywords:** SARS-CoV-2, Omicron variant, 5-ALA, SFC, Antiviral activity

## Abstract

The coronavirus disease 2019 (COVID 19) pandemic continues to pose a threat to global health. The severe acute respiratory syndrome coronavirus 2 (SARS-CoV-2) Omicron variant (B.1.1.529) has spread rapidly worldwide and became dominant in many countries. A natural 5-aminolevulinic acid (5-ALA) with sodium ferrous citrate (SFC) has demonstrated antiviral activity in Wuhan, Alpha, Beta, Gamma, and Delta variants of SARS-CoV-2 infections in vitro. In this study, we report antiviral activity of 5-ALA, 5-ALA with SFC led to IC_50_ of 329 and 765/191, respectively after infection with Omicron variant of SARS-CoV-2 in vitro. Our finding suggests that 5-ALA could be used as antiviral drug candidate to treat Omicron variant infected patients.

## To the Editor,

**Fig. 1:**
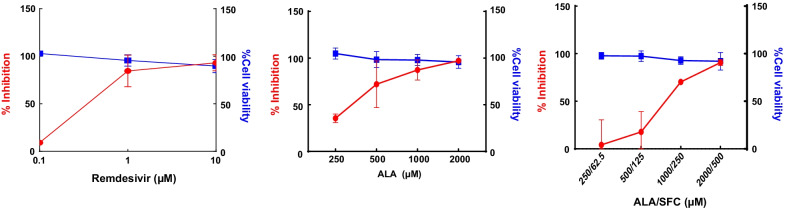
Antiviral effect of remdesivir (**A**) 5-ALA (**B**) and 5ALA with SFC (**C**) against SARS-CoV-2 Omicron variant. Vero E6 cells were pretreated with remdesivir, ALA with and without SFC for 72 h and challenged with SARS-CoV-2. Infected cell supernatants at 48 h pi (MOI 0.02) were quantified by quantitative real time RT-PCR assay. The blue and red lines represent the CC_50_ and IC_50_, respectively; the blue squares represent cell viability (%) and the red circles represent SARS-CoV-2 infection inhibition (%). Data were collected from at least two independent experiments (in replicate) and shown data correspond to the mean ± SD

Multiple severe acute respiratory syndrome coronavirus 2 (SARS-CoV-2) variants have emerged a year and a half from the onset of coronavirus disease 2019 (COVID-19) pandemic. As of February 2022, Omicron variants have been divided into four distinct sub lineages: BA.1, BA.1.1, BA.2, and BA.3 [1–3]. The number of Omicron variant cases has increased in many regions of the world, spreading more easily than previously described SARS-CoV-2 isolates [4]. The Omicron variant has substantial spike protein mutations and is able to escape immune protection elicited by both vaccine and previous infection [5, 6]. Several direct-acting antivirals against COVID-19 have been approved or are under clinical development and can be divided in two categories; small molecules interfering with virus replication machinery, and monoclonal antibodies directed against the spike protein [7]. Aside from improving vaccinations against Omicron and future variants, we must develop new antiviral drugs [8, 9]. Antiviral drugs including remdesivir, molnupiravir, and nirmatrelvir inhibited SARS-CoV-2 Omicron variant infection [7, 10]. A natural amino acid, 5-aminolevulinic acid (5 ALA), is produced from most animals and plants which are present in food. In our previous studies, we reported antiviral activity of 5-ALA with or without sodium ferrous citrate (SFC) against the SARS-CoV-2 Wuhan strain and its variants including Alpha, Beta, Gamma and Delta strains [11, 12]. In this study, we evaluated the antiviral effect of 5-ALA with or without SFC against the SARS-CoV-2 Omicron variant in vitro. Vero E6 cells were treated with remdesivir or 5-ALA with or without SFC for 72 h (h) and then infected with SARS-CoV-2 Omicron variant (TY38-873, BA.1, provided by the Japan National Institute of Infectious Diseases) at a multiplicity of infection of 0.02. After 48 h post infection, the infected cell supernatants were harvested for viral RNA extraction [12, 13]. The SARS-CoV-2 antiviral assay is based on previously established specific quantitative real time PCR (qRT-PCR) [12] using cell supernatant RNA. The antiviral drug effect of remdesivir against the Omicron variant showed an IC_50_ (virus inhibition by 50%) of 0.3 µM (Fig. 1A). 5-ALA, 5-ALA and SFC inhibited SARS-CoV-2 Omicron variant infection in a dose dependent manner with an IC_50_ of 329 and 765/191, respectively in vitro (Fig. 1B, C, Table 1). A cell viability assay was conducted in parallel with the antiviral assay [12, 13] and no cytotoxic effects were observed with CC_50_ (cell survival by 50%) of 5-ALA > 2000 µM and of SFC > 500 µM in Vero E6 cells (Table 1). The Omicron variant which has notable mutations in the receptor binding domain of spike glycoprotein appears to be highly transmissible and less responsive to several of the currently used drugs [14]. Exogenously supplied 5-ALA prompted increased generation of protoporphylin IX (PPIX) and heme inside host cells, likely interfering with interaction of G-quadruplex (G4) structures [15] which inhibited SARS-CoV-2 infection. The G4 structure included in coronaviruses plays a key role in the genome replication/transcription [16]. 5-ALA with SFC is a supplement formulation registered in Japan as a food with functional claims. In a recent clinical study, Japanese patients with COVID-19 who were given 5-ALA and SFC capsules orally experienced a shorter time to recovery than that reported for patients who received only standard care for SARS-CoV-2 infection [17]. Recruitment for clinical trials on the effects of 5-ALA with SFC on COVID-19 outcomes in humans has been completed and the data is now being analyzed (Japan Registry of Clinical Trials CRB 7180001 and 3190006, respectively). Mitochondrial dysfunction has been reported as a cause of disorders in COVID-19 [18]. Given that 5-ALA activates the respiratory chain of mitochondria via heme bio-synthesis, maintenance of mitochondrial function is also expected to play a role in the effect of 5-ALA on the prevention and treatment of COVID and long COVID. In conclusion, we report the antiviral effects of 5-ALA with or without SFC on SARS-CoV-2 Omicron variant in vitro as a potential therapeutic and prophylaxis for COVID-19.

**Table 1:** IC_50_ and CC_50_ values of 5-ALA and 5-ALA with SFC against SARS-CoV-2 Omicron variant

SARS-CoV-2 variants*	Compound	IC_50_ (µM)	CC_50_ (µM)
Wuhan	5-ALA	207	> 2000
	5-ALA/SFC	235/58.7	> 2000/ > 500
Alpha	5-ALA	104	> 2000
	5-ALA/SFC	173/43.2	> 2000/ > 500
Beta	5-ALA	1592	> 2000
	5-ALA/SFC	1311/327.7	> 2000/ > 500
Gamma	5-ALA	> 2000	> 2000
	5-ALA/SFC	1516/379	> 2000/ > 500
Delta	5-ALA	> 2000	> 2000
	5-ALA/SFC	397/99.2	> 2000/ > 500
Omicron	**5-ALA**	**329**	** > 2000**
	**5-ALA/SFC**	**765/191**	** > 2000/ > 500**

IC_50_ and CC_50_ of SARS-CoV-2* variants (Wuhan, Alpha, Beta, Gamma and Delta) was shown in our previous study

In 5-ALA/SFC compound, the ratio of 5-ALA to SFC was fixed as 4:1

*IC*_50_ 50% inhibition concentration, *CC*_50_ 50% cytotoxicity concentration, *5-ALA* 5-aminolevulinic acid, *SFC* sodium ferrous citrate

Bold text indicated IC_50_ and CC_50_ of Omicron variant in this study

## Data Availability

Not applicable.

## Abbreviations

SARS-CoV-2: Severe acute respiratory syndrome coronavirus 2
COVID-19: Coronavirus disease 2019
5-ALA: 5-Aminolevulinic acid
SFC: Sodium ferrous citrate
PPIX: Protoporphyrin IX
qRT-PCR: Quantitative real time reverse-transcription polymerase chain reaction
IC_50_: Virus inhibition by 50%
CC_50_: Cell survival by 50%
G4: G-quadruplex
